# Mathematical Modeling of Locoregional Recurrence Caused by Premalignant Lesions Formed Before Initial Treatment

**DOI:** 10.3389/fonc.2021.743328

**Published:** 2021-10-13

**Authors:** Mitsuaki Takaki, Hiroshi Haeno

**Affiliations:** Department of Computational Biology and Medical Sciences, Graduate School of Frontier Sciences, The University of Tokyo, Chiba, Japan

**Keywords:** mathematical modeling, tumor recurrence, premalignant lesions, stochastic processes, field cancerization

## Abstract

Locoregional recurrence after surgery is a major unresolved issue in cancer treatment. Premalignant lesions are considered a cause of cancer recurrence. A study showed that premalignant lesions surrounding the primary tumor drove a high local cancer recurrence rate after surgery in head and neck cancer. Based on the multistage theory of carcinogenesis, cells harboring an intermediate number of mutations are not cancer cells yet but have a higher risk of becoming cancer than normal cells. This study constructed a mathematical model for cancer initiation and recurrence by combining the Moran and branching processes in which cells require two specific mutations to become malignant. There are three populations in this model: (i) normal cells with no mutation, (ii) premalignant cells with one mutation, and (iii) cancer cells with two mutations. The total number of healthy tissue is kept constant to represent homeostasis, and there is a rare chance of mutation every time a cell divides. If a cancer cell with two mutations arises, the cancer population proliferates, violating the homeostatic balance of the tissue. Once the number of cancer cells reaches a certain size, we conduct computational resection and remove the cancer cell population, keeping the ratio of normal and premalignant cells in the tissue unchanged. After surgery, we considered tissue dynamics and eventually observed the second appearance of cancer cells as recurrence. Consequently, we computationally revealed the conditions where the time to recurrence became short by parameter sensitivity analysis. Particularly, when the premalignant cells’ fitness is higher than normal cells, the proportion of premalignant cells becomes large after the surgical resection. Moreover, the mathematical model was fitted to clinical data on disease-free survival of 1,087 patients in 23 cancer types from the TCGA database. Finally, parameter values of tissue dynamics are estimated for each cancer type, where the likelihood of recurrence can be elucidated. Thus, our approach provides insights into the concept to identify the patients likely to experience recurrence as early as possible.

## Introduction

Locoregional recurrence after surgery appears in many cancer types. About 8% of invasive breast cancer patients exhibited local recurrence after surgical resection with free resection margins (1). In non-small-cell lung cancer, about 25% of patients showed locoregional recurrence after wedge resection (2). In colorectal cancer, over 4% of patients developed locoregional recurrence after surgery (3). To prevent the emergence of recurrent tumors, treatment strategies, such as adjuvant chemotherapy has been examined and improved (4). However, tumor recurrence remains a problem.

A major cause of local recurrence is field cancerization (5–7). Field cancerization was initially defined as the presence of histologically abnormal tissue surrounding primary cancer, but currently, the concept includes the spread of histologically normal but genetically altered cells (5, 8). These cells are prone to be hotbeds for recurrent tumors because they have already accumulated specific cancer-related mutations, and a small number of additional ones is necessary to trigger cancer initiation there. Molecular evidence of field cancerization has been investigated in each tissue (6, 8–10). For example, in breast cancer, microsatellite markers, epigenetic aberrations, and hTERT overexpression have been detected in histologically normal mammary tissues (8). In head and neck cancer, loss of heterozygosity of chromosome 9p was commonly observed in benign squamous hyperplasia (9). In colon cancer patients with Crohn’s ileocolitis, the same mutations of *KRAS*, *CDKN2A*, and *TP53* were observed within neoplasia and non-tumor epithelium (10). Interestingly, locoregional recurrence rates and field cancerization molecular mechanism vary among cancer types. Therefore, understanding field cancerization formation process will contribute to the estimation of the risk of locoregional recurrence and the development of optimal treatment in each tissue.

Theoretical studies have investigated field cancerization impacts on the emergence of recurrent tumors (11–15). Jeon et al. examined the multistage clonal expansion model by employing the Poisson process to consider the effects of premalignant cells on cancer initiation (11). The model was applied to the clinical practice of neoplasia in Barrett’s esophagus. In this study, they succeeded in demonstrating the clinical utility of the model by predicting the long-term impact of ablative treatments on reducing esophageal adenocarcinoma incidence (13). Foo et al. developed a spatial evolutionary framework to study the cancer field effect. They analytically showed the size distribution of histologically undetectable premalignant fields during diagnosis (12). The model was applied to the head and neck cancer and revealed that the patient’s age was a critical predictor of the size and multiplicity of precancerous lesions (14). Although theoretical studies have shed light on field cancerization effects on the emergence of primary and recurrent cancers, the relationship between tissue kinetic parameters and the incidence of recurrent cancers is unclear.

This study developed a novel mathematical model of recurrent tumor evolution. We employed a stochastic process of a multistage model to represent the accumulation of mutations in a tissue, leading to cancer relapse after surgical resection of the first tumor. Particularly, we focused on the relationship between the tissue compositions at the time of surgery and the time until the emergence of recurrent tumors. Our approach provided insights on how to predict the time of recurrence from the tissue dynamics at the time of surgery and how to intervene patients to prevent the recurrence.

## Material and Methods

### Mathematical Model

**Figure 1:**
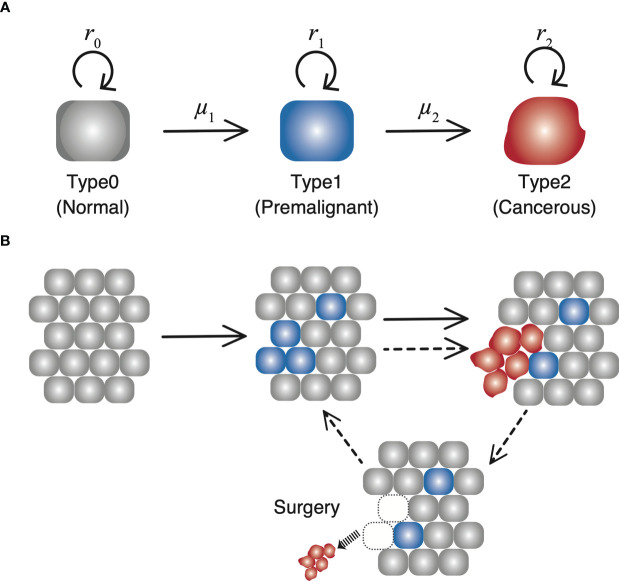
The schematic diagram of our model. **(A)** There are three types of cells with each own mutation rate and fitness in the model. **(B)** In a normal tissue, composed of Type0 and Type1 cells, cell turnover is conducted according to the Moran process, and the number of cells is kept constant. If a Type2 cell emerges, it proliferates unlimitedly over the tissue, and grows up to 10^9^. Once the number reaches 10^9^, all the Type2 cells are resected while the number of Type1 cells in a tissue are preserved. Then the time until the next Type2 population reaches 10^9^ is measured as time of recurrence.

Let us consider the dynamics of three types of cells in a tissue (**Figure 1**). “Type0,” “Type1,” and “Type2” represent normal healthy cells with no mutation, premalignant cells with one cancer-related mutation, and cancer cells with two cancer-related mutations, respectively. We assume that a normal healthy tissue consists of Type0 and Type1 cells performing a turnover of cells with a small probability of a mutation. Moran process is employed to consider the tissue turnover dynamics, where the total number of Type0 and Type1 cells is kept constant as *N* (16). The average turnover time of a whole tissue is defined by *δ* days. Type2 cells are considered as uncontrolled cancer cells proliferating. The branching process is employed to consider the process of Type2 proliferation (17).

Initially, *N* Type0 cells occupy the tissue. There is a rare chance of a mutation every time a cell divides, and a daughter cell may change into a Type1 cell with a mutation rate, *μ*_1_. A cell to be divided in a tissue is selected depending on the fitness of Type0 cells (*r*_0_) and that of Type1 cells (*r*_1_) weighted by the proportion of Type0 and Type1 cells in a tissue. When a Type1 cell divides, a daughter cell may change into a Type2 cell with a mutation rate, *μ*_2_. Once a Type2 cell appears, the cells proliferate indefinitely based on the growth rate of Type2 cells, *r*_2_, ignoring a number restrictions of a tissue unless they go extinct stochastically. In other words, the net growth of Type0 and Type1 cells is zero (equal frequency of cell division and death), while that of Type2 cells is positive. Type0 and Type1 cells consist of a healthy tissue based on the Moran process, so *r*_0_ and *r*_1_ are just parameters to determine which to choose as a dividing cell at the time of a cell turnover. Alternatively, *r*_2_ is the growth rate, which determines the average number of increases in Type2 cells during a unit time. When the number of Type2 cells reaches 10^9^ at the first time, all the Type2 cells are discarded to represent surgical resection, whereas the number of Type1 cells in a tissue is preserved so that the time until the emergence of the recurrent tumor is influenced by the frequency of residual Type1 cells. Since the conversion from the number of cells to the tumor volume is frequently done using the following relationship as 10^9^ cells in a 1 cm^3^ tumor, the time of surgery in this model is conducted when the size of the tumor becomes 1 cm^3^. After the first treatment, the simulation continues until the next Type2 cell appears from the tissue and number reach 10^9^ again, representing the recurrence of the tumor after surgery.

### Simulation Framework

To integrate the Moran process and branching process, we adopted stochastic simulations based on Gillespie’s algorithm (18) as follows: We firstly considered three events: (i) cell turnover in a healthy tissue, (ii) death of a Type2 cell, and (iii) birth of a Type2 cell. The rates of each event at time *t* is given by (i) $\frac{1}{\delta }N$ (ii) *d*_2_ *X*_2_(*t*), and (iii) *r*_2_ *X*_2_(*t*), respectively. Here *d*_2_, *r*_2_, and *X*_2_(*t*) were a death rate, a proliferation rate, and the number of Type2 cells, respectively. Then an average time until one of the three events happens, Δ*T*, is given by


(1) $$\Delta T=\frac{1}{\frac{1}{\delta }N+d_{2}X_{2}\left(t\right)+r_{2}X_{2}\left(t\right)}$$

When the event of cell turnover in a healthy tissue occurs, one of *N* cells is selected as a cell to die, and another cell divides within the time step to complete cell turnover. In detail, there are three possibilities of state transitions in the tissue dynamics: the number of Type1 cells (i) increases by one, (ii) decreases by one, and (iii) does not change. Let us denote the number of Type1 cells by *i*.

First of all, the case (i) occurs through two ways: (a) A Type0 cell dies, and a Type1 cell divides without a mutation; and (b) a Type0 cell dies, and another Type0 cell divides with a mutation to be a Type1 cell. Exceptionally, when a Type0 dies, and a Type1 cell divides with a mutation to be a Type2 cell, an additional selection of a cell to divide is done because a Type2 cell cannot reside in a normal tissue under the assumption of the model. In this situation, if a Type1 cell is selected to divide without a mutation, the number of Type1 cells increases by one. The probabilities of these three events are given by $\frac{N-i}{N}\cdot \frac{r_{1}i\left(1-\mu_{2}\right)}{F},\;\frac{N-i}{N}\cdot \frac{r_{0}\left(N-i\right){µ}_{1}}{F},$ and $\frac{N-i}{N}\cdot \frac{r_{1}i{µ}_{2}c_{1}}{F}$ respectively. Here *F* = *r*_0_ (*N* – *i*) + *r*_1_ *i* is a scaling factor for the probability to be chosen for a dividing cell and $c_{1}=\frac{r_{1}i}{r_{0}\left(N-i\right)+r_{1}i}$ is the probability that a Type1 cell is selected to divide in an additional round after a mutation of a Type1 cell to be a Type2 cell. The probability that a Type0 cell is selected to die is given by $\frac{N-i}{N}$ . Taken together, the transition probability that the number of Type1 cells increases by one is given by


(2) $$\text{Pr}\left[i\to i+1\right]=\frac{r_{0}\left(N-i\right){\text{µ}}_{1}+r_{1}i\left(1-{\text{µ}}_{2}+{\text{µ}}_{2}c_{1}\right)}{F}\cdot \frac{N-i}{N}$$

Secondly, the case (ii) occurs in such a way that a Type1 cell dies and a Type0 cell divides without a mutation. Exceptionally, when a Type1 cell dies, and another Type1 cell divides with a mutation to be a Type2 cell, an additional selection for a cell division is done. In this case, if a Type0 cell is selected for the additional cell division, the number of Type1 cells decreases by one. The probabilities of the two events are given by $\frac{i}{N}\cdot \frac{r_{0}\left(N-i\right)\left(1-{µ}_{1}\right)}{F}$ and $\frac{i}{N}\cdot \frac{r_{1}i{µ}_{2}c_{0}}{F}$ and $c_{0}=\frac{r_{0}\left(N-i\right)}{r_{0}\left(N-i\right)+r_{1}i}$ is the probability that a Type0 cell is selected to divide in an additional round after a mutation of a Type1 cell to be a Type2 cell. The probability that a Type1 cell is selected to die is given by $\frac{i}{N}$ . Taken together, the transition probability that the number of Type1 cells decrease by one is given by


(3) $$\text{Pr}\left[i\to i-1\right]=\frac{r_{0}\left(N-i\right)(1-{\text{µ}}_{1})+r_{1}i{\text{µ}}_{2}c_{0}}{F}\cdot \frac{i}{N}$$

Finally, the probability that the number of Type1 does not change [case (iii)] is given by


(4) Pr[i→i]=1−Pr[i→i+1]−Pr[i→i−1]

In summary, the time of one step in simulations is calculated using Eq. (1), and in one step, one of the following three processes occurs: (i) cell turnover in a tissue, (ii) the death of a Type2 cell, or (iii) the birth of a Type2 cell. When case (i) happens, there are three possibilities in tissue dynamics. The number of type1 cells increases by one, decreases by one, or does not change. Initially, all the cells are Type0. Once the number of Type2 cells reaches 10^9^, computational surgical resection to set the number of Type2 cells to be 0 again will be conducted. After that, the time until the number of Type2 cells reaches 10^9^ again is measured as recurrence time.

### Deterministic Approximation of Type2 Growth

As for the calculation of the Type2 growth, we assumed that when the number of cells is small, the stochastic effect should be considered. When the number of Type2 cells exceed twice as large as the size of the normal tissue, 2*N*, growth can be regarded as a deterministic process. Then the time duration from when the number of Type2 cells is 2*N* to 10^9^, Δ*t_s_*, is given by


(5) $$\Delta t_{s}=\left(r_{2}-d_{2}\right)\ln\left(\frac{10^{9}}{2N}\right)$$

During Δ*t_s_*, tissue dynamics to reflect the cell turnover is conducted.

### Clinical Data

The data used in our analysis were downloaded from TCGA Pan-Cancer Clinical Data Resource provided in the previous publication (19). We adopted the data of disease-free intervals from 23 cancer types. Data processing was performed on Excel.

### Survival Time Analysis

Disease-free survival of clinical data were calculated using the Kaplan–Meier method from disease-free intervals mentioned in *Clinical Data* section. In this study, disease-free interval is defined as the survival time without cancer recurrence of each patient, which corresponds to the time to recurrence of each simulation trial. Disease-free survivals *in silico* were then calculated from that.

### Simulation and Statistical Analysis

The whole process of our model was conducted on C++. Parameter optimization was conducted using the Nelder–Mead method on R (version 3.6.2). The survival time analysis was conducted on Prism (version 8.4.3).

## Results

### Three Patterns of Cancer Initiation

**Figure 2:**
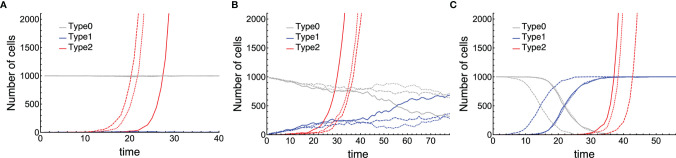
Three patterns of cancer initiation. Gray, blue, and red curves describe Type0, Type1, and Type2 cells, respectively (the full growth dynamics are not shown). Each panel contains three trials of the same parameter sets distinguished by the type of lines: Joined, dashed, and long-dashed. Cancer initiates from: **(A)** almost no Type1 cells (*i* ≤ 0.1*N)*; **(B)** moderate number of Type1 cells (0.1*N* < *i* ≤ 0.9*N*); and **(C)** occupied Type1 cells (*i* > 0.9*N*). Parameter values used are *N* = 1,000, *δ* = 1.0, *r*_0_ = 1.0, *r*_1_ = 0.75, *r*_2_ = 1.5, *μ*_1_ = 0.001, and *μ*_2_ = 0.1 for **(A)**; *N* = 1,000, *δ* = 1.0, *r*_0_ = 1.0, *r*_1_ = 1.0, *r*_2_ =1.2, *μ*_1_ = 0.01, and *μ*_2_ = 0.01 for **(B)**; and *N* = 1,000, *δ* = 1.0, *r*_0_ = 1.0, *r*_1_ = 1.5, *r*_2_ = 1.5, *μ*_1_ = 3.16 ∙ 10^–4^, and *μ*_2_ = 3.16 ∙ 10^–4^ for **(C)**.

First of all, we conducted stochastic simulations of the model for the initial cancer progression, and the time courses of three populations: Type0, Type1, and Type2 were shown (**Figure 2**). We classified the tissue dynamics until the emergence of Type2 cells into three patterns. When Type1 cells had less fitness than Type0 cells, sporadic cancer initiation from a tissue dominated by Type0 cells could be observed (**Figure 2A**). In this case, Type1 cells could not spread in a normal tissue, and cancer initiation depended on two sequential mutations in one Type1 cell. After surgical resection of the first Type2 lineage, the time to recurrence would be almost the same as that of the first cancer initiation because the frequency of Type1 cells in a tissue was almost the same as the initial condition. When the fitness of Type1 cells was as high as that of Type0 cells, cancer initiation in a moderate frequency of Type1 cells could be observed (**Figure 2B**). In this case, the time to recurrence could be faster than that of the first cancer initiation because the proportion of Type1 cells in a tissue was larger than that in the initial condition. When Type1 cells had much higher fitness than Type0 cells, multiple cancer initiations from a Type2-dominated tissue could be observed (**Figure 2C**). In this case, the recurrence of tumors happened easily. From these results, we found that different situations of Type1 cells at the time of cancer initiation were considered to influence the difficulty of recurrence, and they could be classified by parameter regions.

### Parameter Dependency

**Figure 3:**
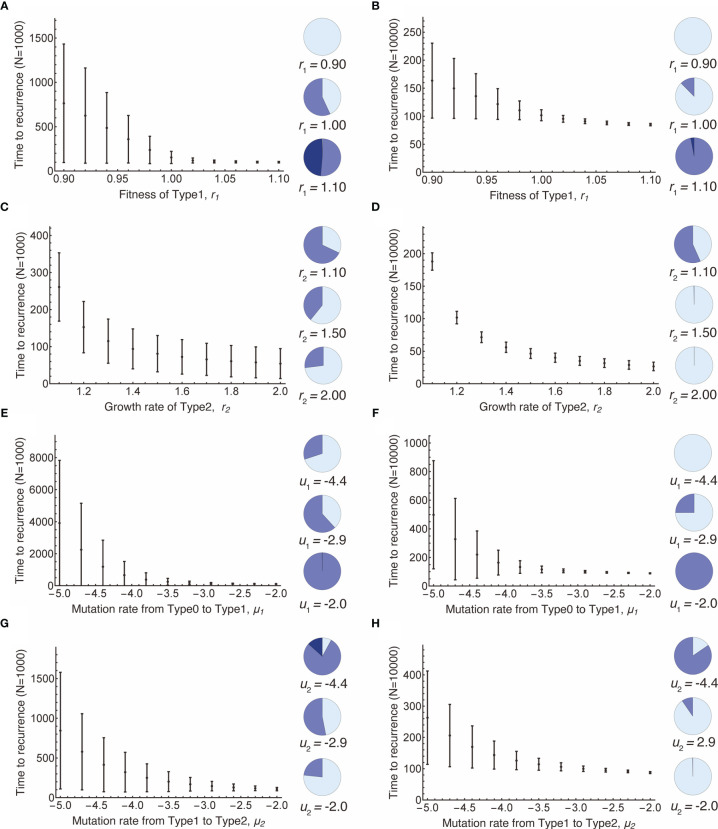
Parameter dependence on recurrence time. Mean values obtained from the simulations are shown by dots, and standard deviations are indicated by bars. Pie charts in the panels indicate the proportion of Type1 cells in normal tissue at the first treatment. Light blue, blue, dark blue represent small (*i* ≤ 0.1*N*), intermediate (0.1*N* < *i* ≤ 0.9*N*), and large (*i* > 0.9*N*) proportion of Type1 cells, respectively. Standard parameter values used in **(A–H)** are *δ* = 1.0, *r*_0_ = 1.0, *r*_1_ = 1.0, *r*_2_ = 1.2, μ_1_ = 0.001, μ_2_ = 0.001; and *N* = 1,000 in **(A, C, E, G)**; and *N* = 10,000 in **(B, D, F, H)**.

Next, we examined the time to recurrence after surgical resection and the proportion of premalignant (Type1) lesions at the time of surgery in a vast parameter range (**Figure 3**). The mean recurrence time became shorter as the fitness of Type1 cells increased because higher fitness enabled Type1 cells to dominate the normal tissue, which facilitated the emergence of recurrent cancer (Type2) (**Figures 3A, B**). When the size of the normal tissue is small, the effect of fitness advantage on the proportion of Type1 cells in a tissue became large (**Figures 3A, B**). **Figures 3C, D** showed that recurrence time became shorter when the growth rate of Type2 cells was large. Compared to the case where the fitness of Type1 was large, the early recurrence occurred from the small proportion of Type1 cells in a tissue (**Figures 3C, D**). High mutation rates accelerated the time of recurrence (**Figures 3E–H**). A higher mutation rate from Type0 to Type1 made the proportion of Type1 cells larger (**Figures 3E, F**), while a higher mutation rate from Type1 to Type2 made proportion smaller (**Figures 3G, H**). Furthermore, when the size of normal tissues became large, the time to recurrence became short, and the variation became small (**Figures 3B, D, F, H**).

### Relationship Between the Proportion of Type1 Cells and Time to Recurrence

**Figure 4:**
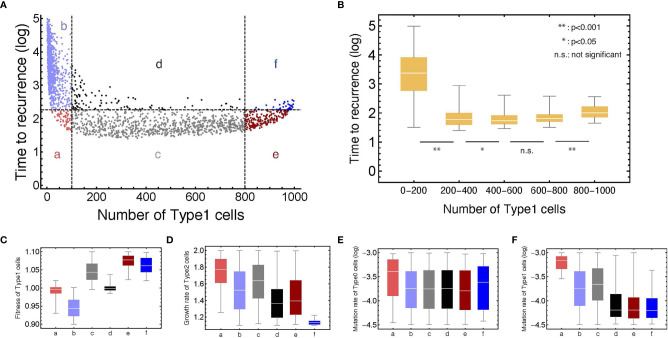
The relationship between the number of Type1 cells in the normal tissue at the first treatment and time to recurrence with various parameter values. **(A)** Points are generated by the simulations with parameter sets which are randomly chosen from: 0.90 < *r*_1_ < 1.10, 1.10 < *r*_2_ < 1.20, 10^–4.5^ < *μ*_1_ < 10^–3.0^, and 10^–4.5^ < *μ*_2_ < 10^–3.0^, respectively. The plots are categorized into six areas with the median of time to recurrence. Note that *δ* = 1.0, *r*_0_ = 1.0, and *N* = 1,000. **(B)** Box plots show the distributions of time to recurrence from different ranges of the Type1 proportion in a tissue at the time of the first treatment. **(C–F)** Box plots represent the parameter distributions in each category determined in panel **(A)**. Each bar corresponds to the area notation in panel **(A)**.

To investigate the relationship between the proportion of Type1 cells during initial treatment and time to recurrence comprehensively, we conducted computational simulations with parameter sets randomly picked (**Figure 4A**). Additionally, we did 1,000 runs of stochastic simulations with the same parameter set to obtain each point. A total of 1,200 parameter combinations were examined.

We confirmed that recurrence time was significantly different among the proportion of Type1 cells during the first treatment (**Figure 4B**). It would be intuitive that the time to recurrence became long when the proportion of Type1 cells was very small (between 0 and 0.2 of a tissue). Interestingly, the proportion of Type1 cells that minimize recurrence time was not the largest group (between 0.8 and 1.0 of a tissue), but the moderate group (**Figure 4B**). This result showed that patients with a moderate number of premalignant cells (Type1) have a risk of shorter recurrence time in many cases. When we investigated the characteristics of parameter values in each category (**Figures 4C–F**), we found that the fitness of Type1 cells was lower, and their mutation rate was higher in areas a and b than those in areas e and f (**Figures 4C, F**). These results suggested that Type1 cells could occupy the normal tissue before the first treatment when Type1 cells could spread rapidly and were hardly mutated to be Type2 cells. The mutation rate of Type0 cells did not affect the proportion of Type1 cells at the first treatment (**Figure 4E**). Points with time to recurrence more than 10^3^ only resided in area b, indicating that there was no parameter set that could realize both conditions of a large proportion of Type1 cells at the time of first treatment and a long recurrence time (**Figure 4A**). In area a, time to recurrence was short despite small premalignant cells (Type1). In that case, the fitness of Type1 cells was almost neutral, and the mutation rate of Type1 cells and the growth rate of Type2 cells were relatively high (**Figures 4C, D, F**). In area f, recurrence was relatively long, although the normal tissue was occupied by premalignant cells (Type1). In that case, the growth rate of Type 2 cells was extremely small (**Figure 4D**). Mutation rates of areas d, e, and f were almost the same, and their difference was generated by the fitness of Type1 cells and the growth rate of Type2 cells (**Figures 4C, D**).

### Fitting to Clinical Data of Time to Recurrence

**Figure 5:**
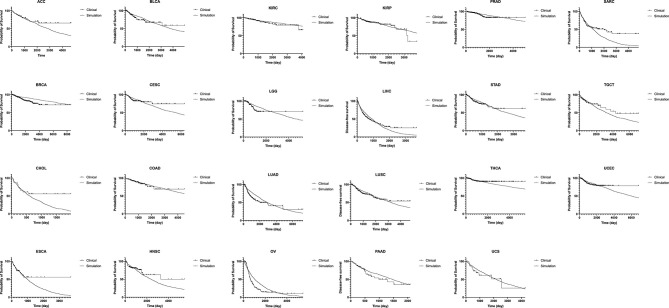
Fitting of our model to clinical data of disease-free survival in 23 cancer types. Results of disease-free survival *in silico* (thin curve) are fitted to that of published clinical data in 23 cancer types (thick curve). A thousand runs of stochastic simulations with a single parameter combination for each cancer type are performed. The parameter values used for each panel is listed in **Table 1**. ACC, adrenocortical carcinoma; BLCA, bladder urothelial carcinoma; BRCA, breast invasive carcinoma; CESC, cervical squamous cell carcinoma and endocervical adenocarcinoma; CHOL, cholangiocarcinoma; COAD, colon adenocarcinoma; ESCA, esophageal carcinoma; HNSC, head and neck squamous cell carcinoma; KIRC, kidney renal clear cell carcinoma; KIRP, kidney renal papillary cell carcinoma; LGG, brain lower grade glioma; LIHC, liver hepatocellular carcinoma; LUAD, lung adenocarcinoma; LUSC, lung squamous cell carcinoma; OV, ovarian serous cystadenocarcinoma; PAAD, pancreatic adenocarcinoma; PRAD, prostate adenocarcinoma; SARC, sarcoma; STAD, stomach adenocarcinoma; TGCT, testicular germ cell tumors; THCA, thyroid carcinoma; UCEC, uterine corpus endometrial carcinoma; UCS, uterine carcinoma.

Results of recurrence time *in silico* were fitted to published clinical data of disease-free survivals in 23 cancer types (**Figure 5** and **Table 1**) (19). A thousand runs of stochastic simulations with a single parameter combination for each cancer type were conducted. The sum of squared logarithmic residuals (log-SSR) between outputs *in silico* and five data points extracted from clinical data was calculated. A set of the five data points was when 20, 40, 60, 80, and 100% of patients experienced a recurrence. We then investigated the parameter sets that could minimize log-SSR for each cancer type (**Table 1**), and depicted the survival curves with the estimated parameters (**Figure 5**). We also conducted a log-rank test between the curves of clinical and simulated data (**Table 1**). In most clinical data, we could find the optimal parameter sets, and with these parameters, significant differences were not observed between simulation results and clinical outcomes. However, in some cancer types (BRCA, CHOL, LUAD, OV, SARC, and THCA), significant deviations were observed (p < 0.05). Notably, the fitness of Type1 cells was lower than that of Type0 cells, 1.0, among most cancer types, indicating a cancer-related mutation tends to be disadvantageous before the emergence of cancer cells (Type2). Mutation rates were distributed around 10^−3.6^ for almost all cancer types. Alternatively, the growth rates of Type2 were widely distributed.

**Table 1:** Estimated parameters and p-values by fitting the outputs from our simulations to clinical data.

Cancer type	*r*_1_	*r*_2_	Log_10_ *μ*	log-SSR	p-value
ACC	0.916	1.62	−3.61	0.272	0.2008
BLCA	0.908	1.43	−3.60	0.717	0.4658
BRCA	0.922	1.52	−4.02	0.294	<0.0001
CESC	0.905	1.36	−3.63	0.815	0.8958
CHOL	0.964	1.52	−3.43	0.113	0.0272
COAD	0.924	1.40	−3.71	0.564	0.5966
ESCA	0.934	1.60	−3.42	0.128	0.3458
HNSC	0.914	1.58	−3.56	0.926	0.3446
KIRC	0.920	1.27	−3.77	0.314	0.3945
KIRP	0.908	1.38	−3.62	0.981	0.6651
LGG	0.905	1.35	−3.62	0.0312	0.0803
LIHC	0.962	1.72	−3.54	0.647	0.8949
LUAD	0.920	1.62	−3.62	1.52	0.0039
LUSC	0.904	1.43	−3.55	0.588	0.4501
OV	0.905	1.56	−3.37	0.604	<0.0001
PAAD	0.918	1.54	−3.44	0.139	0.3649
PRAD	0.913	1.34	−3.80	0.165	0.1207
SARC	0.930	1.51	−3.42	1.05	0.0036
STAD	0.917	1.59	−3.59	0.432	0.3859
TGCT	0.916	1.61	−3.63	1.68	0.4146
THCA	1.04	1.10	−3.31	7.74	<0.0001
UCEC	0.909	1.33	−3.65	0.168	0.4777
UCS	0.904	1.53	−3.49	0.633	0.6923

ACC, adrenocortical carcinoma; BLCA, bladder urothelial carcinoma; BRCA, breast invasive carcinoma; CESC, cervical squamous cell carcinoma and endocervical adenocarcinoma; CHOL, cholangiocarcinoma; COAD, colon adenocarcinoma; ESCA, esophageal carcinoma; HNSC, head and neck squamous cell carcinoma; KIRC, kidney renal clear cell carcinoma; KIRP, kidney renal papillary cell carcinoma; LGG, brain lower grade glioma; LIHC, liver hepatocellular carcinoma; LUAD, lung adenocarcinoma; LUSC, lung squamous cell carcinoma; OV, ovarian serous cystadenocarcinoma; PAAD, pancreatic adenocarcinoma; PRAD, prostate adenocarcinoma; SARC, sarcoma; STAD, stomach adenocarcinoma; TGCT, testicular germ cell tumors; THCA, thyroid carcinoma; UCEC, uterine corpus endometrial carcinoma; UCS, uterine carcinoma.

## Discussion

In this study, we constructed a mathematical model that could describe cell population dynamics in both normal tissue and cancer tissues. We revealed the relationship between the proportion of premalignant cells and recurrence time (**Figures 3** and **4**). Importantly, we found that recurrence time became shorter when the mutation rate or growth rate of cancer cells was large, while the time became longer when the fitness of premalignant cells or growth rate of cancer cells was low (**Figure 4**). Moreover, we successfully estimated the characteristic parameter sets of the computational model by fitting the model results to the clinical data of disease-free survival in each cancer type (**Figure 5** and **Table 1**). This study is the first attempt to quantitatively predict recurrence time after the first treatment in various cancer types with a mathematical model by considering the effect of premalignant cells in a healthy tissue.

This model successfully reproduced the disease-free survivals in 17 out of 23 cancer types (**Figure 5** and **Table 1**). Notably, the estimated fitness values of premalignant cells (*r*_1_) were less than those of normal cells in many cancer types (**Table 1**). According to the analysis on how the proportion of premalignant cells depended on their fitness (**Figure 4C**), the characteristics of those cancers residing in area b in **Figure 4A** suggest the small abundance of premalignant cells during the first treatment. Therefore, the efforts to find and eradicate the residual premalignant lesions in a normal tissue after the first treatment may be inefficient; rather, the suppression of the emergence of new premalignant cells from the normal cells by adjuvant therapy should be recommended. In most cancer types, the fitting tends to work for the early reduction of the disease-free survivals and not for the long tail of the survivals (**Figure 5**). Because the estimated parameters of the low fitness of premalignant cells (*r*_1_) indicate that recurrence arises from the almost non-mutated tissue, it implies that the deviance recurrence time in the same cancer type is caused by variations of mutation rates or efficiency of adjuvant therapy among patients, not incorporated into the model. It suggests the importance of identifying a biomarker to classify recurrence-prone patients (20).

For the model’s simplicity, we prepared only one population for intermediate cell type as premalignant cells. However, the multistage theory suggested more than two steps to generate a cancer cell from a normal cell (21). This restriction resulted in the simple tendency of the survival curves from the model and failure to fit the long tail of clinical survival curves (**Figure 5**). With multiple stages of premalignant cells in the model, the premalignant cells after the first treatment have several mutational distances to recurrence, which may generate multiple inclinations of the survival curves. In contrast, the number of mutations required to be a cancer cell varies in each patient, even in the same cancer type, so that it was difficult to determine it accurately for each cancer type. This simple model structure had the abovementioned weakness but still could imply that the single-intermediate population might be enough to reproduce the data of well-fitted cancer types, while more populations would be required for the others. We also adopted a spatially homogeneous process, though a spatial process can contain detailed information, such as molecular mechanisms of field cancerization and cell competition. Note that this study focused on constructing the basic mathematical model extensible for various types of cancer to quantitatively predict recurrence time after the first treatment by considering the effect of premalignant cells. Molecular mechanisms vary among cancer types, and cell competition can be regarded as dynamics based on the fitness and the number of the cells. The simple model structure enabled us to analyze the various types of cancer by uniformed parameters, fitness, and mutation rate. This was the first attempt, and even at the current stage, we obtained many new insights. A spatial structure and additional intermediate populations optimized for each cancer type would be a possible future extension of the model.

Conclusively, this model suggests special care of recurrence in the clinic when the fitness of premalignant cells and the growth rate of recurrent tumors is high. Furthermore, this approach can be extended to explore the deviance of recurrence rates among cancer types by introducing the variations of mutational stages and standard adjuvant therapies in each cancer according to growing knowledge.

## Data Availability Statement

The original contributions presented in the study are included in the article/supplementary material. Further inquiries can be directed to the corresponding author.

## Author Contributions

HH supervised the work. MT performed theoretical analysis. MT and HH wrote manuscript. All authors contributed to the article and approved the submitted version.

## Funding

The work is supported by National Cancer Center Research and Development Fund (2021A-7), a research grant from SRL, H.U Group Research Institute, and JSPS KAKENHI Grant Number 20J22335. The funder was not involved in the study design, collection, analysis, interpretation of data, the writing of this article or the decision to submit it for publication.

## Conflict of Interest

The authors declare that the research was conducted in the absence of any commercial or financial relationships that could be construed as a potential conflict of interest.

## Publisher’s Note

All claims expressed in this article are solely those of the authors and do not necessarily represent those of their affiliated organizations, or those of the publisher, the editors and the reviewers. Any product that may be evaluated in this article, or claim that may be made by its manufacturer, is not guaranteed or endorsed by the publisher.
